# Supplementary material for: Threatening language detection from Urdu data with deep sequential model
Source: PLoS One. 2024 Jun 6;19(6):e0290915. doi: 10.1371/journal.pone.0290915 (PMC11156278; doi:10.1371/journal.pone.0290915)
Supplement: Attachment — Submitted filename: PLOS One Comments and solutions.docx [file pone.0290915.s001.docx]

**Additional Editor Comments and solutions**

Comment #1. The novelty of the paper is not justified. Need to benchmark the results with other

Datasets and Baes line Algorithms.

Solution:	in the revised MS we performed additional experiments in order to benchmark our results. We used additional dataset and additional models for experiments. After experiment the result figure show that the proposed LSTM model where data augmentation was used to increase data instances super seeds the other models results. Please refer to table 6.

Comment # 2. The figure of the paper is not clear and up to the mark. Organization of the paper

Need to improve.

Solution:	in the revised Version we re-sketched the blur figures. In the revised version we tried to present the article in more organized way. We adjusted all sections in integrated manner. We hope that the new modification will now meet the reviewer requirements. Please refer to figure 1.

Comment # 3. It is desired to discuss the model complexity of the proposed model and the

baseline models. This would show their efficiency in terms of runtime and space complexity.

Solution:	in the revised MS we discussed the model complexity of the proposed and baseline models as desired by the worthy editor is added in section 5.3.

Comment #4. I suggest the authors give a problem statement or use case in Methodology so as

to help readers understand how to make a recommendation based on the dataset.

Solution:	yes we agree that the previous version lacks problem statement. Now in the revised version we added problem statement in the last para of introduction, so that reader may understand the problem and to justify the contributions.

Comment#5.	I was somewhat confused about the motivations described in the introduction. I

Suggest the authors show the detailed process of parameters. However, the proposed method shows good performance. I also suggest the authors to adding a part of applications to discuss the accurately identifying Threatening Language Detection based the proposed method and obtained results.

Solution:	In the revised version motivations are re written in order to remove the ambiguity. In the result section we added sufficient details regarding the parameters used by the proposed LSTM model. In the result analysis section now we clearly mentioned the key feature through which the proposed model superseded the existing models. We hope that the new addition will now meet the editor requirements. The parameter adopted for the proposed model is provided in table 3.

**Reviewer # 1 comments and solutions**

I will recommend this manuscript a minor revision to reorganize.

We are very thankful for suggesting minor revisions

Comment#1.	It's a well written introductory section. However, in the later section, I cannot see

a similar trend.

Solution:	Yes we agree the later section of introduction lacks integration. In the revised version we tried to present the article in more organized way. We adjusted all sections in integrated manner. We hope that the new modification will now meet the reviewer requirements.

Comment#2. There are some clear constraints in the introductory section on whether a mixed-

Method strategy can be used. I would also recommend that the dissertation be focused on current shortcomings and differences.

Solution:	The current study represents threating text detection using LSTM where data augmentation was used to increase data instances. The proposed method showed significant improvement in the result. Moreover in the revised version we also added limitations and shortcoming of the current study and also added future directions.

Comment#3. The scope and importance of research is not adequately clarified. The simple

Reader does not understand why this subject is important and what is the significance of new research results on such real-world applications, as they appear in our everyday lives.

Solution:	yes we agree that in the previous version the scope and significance was not explained in detail. However now in the revised version we added significance of the proposed approach in the everyday life with suitable examples and justification

Comment#4. There is no contrast of the latest approach introduced to other current methods in

the results section (unless I have ignored).

Solution:	In the revised version we have compared result with sate of the art approaches and results showed significance of the proposed LSTM approach where data augmentation was used to increase data instances.

Comment #5. Add these references in your updated manuscript:

• K. M. Hasib, M. R. Islam, S. Sakib, M. A. Akbar, I. Razzak and M. S. Alam, "Depression Detection From Social Networks Data Based on Machine Learning and Deep Learning Techniques: An Interrogative Survey," in IEEE Transactions on Computational Social Systems, vol. 10, no. 4, pp. 1568-1586, Aug. 2023, doi: 10.1109/TCSS.2023.3263128.
• Hasib, Khan Md, Nurul Akter Towhid, Kazi Omar Faruk, Jubayer Al Mahmud, and M. F. Mridha. "Strategies for enhancing the performance of news article classification in Bangla: Handling imbalance and interpretation." Engineering Applications of Artificial Intelligence 125 (2023): 106688.
• K. M. Hasib, A. Tanzim, J. Shin, K. O. Faruk, J. A. Mahmud and M. F. Mridha, "BMNet-5: A Novel Approach of Neural Network to Classify the Genre of Bengali Music Based on Audio Features," in IEEE Access, vol. 10, pp. 108545-108563, 2022, doi: 10.1109/ACCESS.2022.3213818.

Solution:	Many thanks for suggesting the above references. In the revised version we cited and added the above mentioned references.

**Reviewer #2 comments and solutions:**

The paper represents threating text detection using LSTM where data augmentation was used to increase data instances. The proposed method showed significant improvement in the result. However, the authors contributed two important resources such as vocabulary and stop word list for Urdu language.

Comment #1.	As the authors are using sequential models like LSTM, I think the stop word

removal was not required. At least, authors should see the change in the result without removing stop words.

**Solution:**	Many thanks for pointing this shortcoming in experiments. Now in the revised version we have performed experiments without removing the stop words.

Comment # 2. Please rewrite some sentences in abstract and text where there is lack of clarity.

Solution:	In the revised version we have added some new sentences to clarify and enhance existing abstract as suggested by the reviewer
