# Supplementary material for: Threatening language detection from Urdu data with deep sequential model
Source: PLoS One. 2024 Jun 6;19(6):e0290915. doi: 10.1371/journal.pone.0290915 (PMC11156278; doi:10.1371/journal.pone.0290915)
Supplement: Attachment — Submitted filename: Response to Reviewers.docx [file pone.0290915.s002.docx]

**Comments and solutions**

Reviewer #1: I will recommend a minor revision to reorganize the paper.

**1. It's a well written introductory section. However, in the later section, I cannot see a similar trend.**

**Solution:** In the revised draft, we have made concerted efforts to enhance clarity throughout the remaining sections. We have meticulously reviewed and either removed or rephrased any statements that were deemed irrelevant or ambiguous. Our aim was to ensure that the revised draft is more concise, coherent, and focused. We are optimistic that these revisions will address any concerns raised by the esteemed reviewer and lead to the approval of our manuscript. Thank you for your valuable feedback.

**2. There are some clear constraints in the introductory section on whether a mixed-method strategy can be used. I would also recommend that the dissertation be focused on current shortcomings and differences**.

**Solution:** In the revised version, we have incorporated an analysis of current shortcomings and differences in the introduction section. We believe that by acknowledging and addressing these challenges, our work contributes significantly to advancing the field of Urdu language processing and threat detection, thereby meeting the reviewer's expectations for a comprehensive assessment of the current landscape.

**3. The scope and importance of research is not adequately clarified. The simple reader does not understand why this subject is important and what is the significance of new research results on such real-world applications, as they appear in our everyday lives.**

**Solution:** The introduction section now explicitly outlines the scope and importance of the current work. It aims to tackle the challenges in Urdu language processing for threat detection on social media platforms. By introducing advanced preprocessing techniques and leveraging deep sequential models like Long Short-Term Memory (LSTM) units, the research endeavors to improve the accuracy of threat identification in Urdu textual data. This effort is vital for enhancing safety in online spaces by effectively mitigating threats across various social media platforms in Urdu.

**4. There is no contrast of the latest approach introduced to other current methods in the results section (unless I have ignored).**

**Solution:** Thank you for your feedback. We acknowledge the need to provide a clearer contrast between our latest approach and other current methods in the results section. Indeed, Table 6 offers a methodology comparison where we compared our approach with SVM, CNN, LR, and MLP.

Furthermore, we appreciate your insights into future research directions. We are committed to advancing the field and addressing any remaining limitations. In our future work, we plan to explore ensemble learning techniques, incorporate domain-specific knowledge, address imbalanced data issues, and extend our approach to other languages and platforms. These avenues of investigation will enrich our understanding and contribute to the continued progress of threat detection in online environments. Thank you for bringing these points to our attention, and we will incorporate them into our revisions accordingly.
